# The Effect of Midyear Report Cards on Colonoscopy Quality Measures

**DOI:** 10.1155/2019/4276520

**Published:** 2019-07-22

**Authors:** Kunal Suradkar, Benjamin Lebwohl, Ravi P. Kiran, Steven Lee-Kong

**Affiliations:** ^1^Division of Colorectal Surgery, Columbia University Medical Center/NewYork-Presbyterian Hospital, USA; ^2^Division of Gastroenterology, Columbia University Medical Center/NewYork-Presbyterian Hospital, USA

## Abstract

**Introduction:**

Since 2011, our institution has distributed annual reports, in June, to providers with personalized data regarding adenoma detection rate (ADR), colonoscope withdrawal time (CW), and cecal intubation (CI) rate, using standardized reporting systems. We examined the impact of distribution of individualized reports at the midpoint of each year on colonoscopy outcomes in the latter half of each year.

**Methods:**

Providers with endoscopy privileges, performing ≥20 colonoscopies/year, at our center throughout a five-year period (2011-2015) were included. The three metrics recorded and reported were ADR, CW, and CI using standard benchmark rates. The mean values of each metric from January through June (1^st^ half) and July through December (2^nd^ half) were calculated. Curve estimation test was used to determine the significance of ADR in the respective time period.

**Results:**

Fifteen providers were eligible for the study. Collective ADR in the 1^st^ half of all years was 26.9% and in the second half of all years was 28.1% (*p* = 0.476). CW for all years was more than 9 minutes while CI was above 90% for all providers. There was no significant increase in the CI and CW during the 5-year study period. Overall, ADR increased from 26.43% (2011) to 33.47% (2015) (*p* = 0.137). When examining ADR during each of the 12 months following the June report cards, there was no month-to-month trend observed (*p* = 0.893).

**Conclusion:**

Endoscopists at our institutions met/exceeded the quality metrics in the first half of each year from the beginning of the study. Routine reporting may maintain, but not improve, outcomes. Long-term studies to determine if periodic feedback to endoscopists improves the quality of endoscopy as per national standards for detection of early colorectal cancers are required.

## 1. Introduction

Colonoscopy is widely used in the diagnosis and treatment of colon disorders. It now has a primary role in the detection and prevention of colorectal cancer (CRC), the third leading cause of cancer death in the United States [1]. The American College of Gastroenterology (ACG) and American Society for Gastrointestinal Endoscopy (ASGE) initially published colonoscopy quality guidelines in 1988 and updated them most recently in January 2015 [2–4]. Polypectomy reduces the incidence and mortality from colorectal cancer. The reduction in colorectal cancer death rates has decreased by improving the access to and use of screening and standard treatment in all populations [5].

The ability to reduce the incidence of colorectal cancer using colonoscopy is dependent on the removal of adenomas and hence depends on the operator [6]. Suboptimal performance of colonoscopy by some endoscopists, as evidenced by variable performance, is an obstacle to colonoscopy's ability to provide protection against incident colorectal cancers [7]. Concerned about the miss rate, despite being low, for cancers and adenomatous polyps, high-quality examination was suggested to ensure the detection and removal of all neoplastic lesions [8, 9].

Reducing the variation in quality is now considered an important priority for colonoscopy practice. So as to improve an individual endoscopist's adenoma detection by targeting operator technique or improving preparation quality, quality metrics such as adenoma detection rate (ADR), colonoscope withdrawal time (CW), and cecal intubation (CI) were introduced [10–13].

Some centers send out personalized reports to endoscopists with the details of these indicators [14–16]. At our institution, these reports are distributed annually, in June of each year which includes the adenoma detection rate (ADR), colonoscope withdrawal time (CW), and cecal intubation (CI) rate, using standardized digital reporting systems.

We aimed to investigate whether the distribution of individualized reports of colonoscopy quality metrics at the midpoint of each year improves colonoscopy outcomes in the latter half of each year.

## 2. Methods

This was a retrospective cohort study of endoscopists performing colonoscopies at a single academic medical center (NewYork Presbyterian Hospital, Columbia University Medical Center). This study was approved by our Institutional Review Board.

In June 2011, our institution distributed the first colonoscopy quality report card to each endoscopist detailing his/her ADR, CI, and CW, as well as the institutional mean ADR using standardized digital reporting systems, following an Annual Quality Improvement conference held annually. The deidentified data are presented at the annual meeting and then reports are sent to individual endoscopists [13].

For the purposes of our study, providers with endoscopy privileges at our institution throughout the five-year period spanning 2011-2015 were included. Providers who performed ≥20 colonoscopies per year were included. The three metrics recorded and reported on were ADR, CW, and CI. National standard benchmark rates for these metrics were considered when evaluating providers as follows [7]:

1. Adenoma detection rate, overall 20%: male patients 25% and female patients 15%
2. Colonoscope withdrawal time: >6 minutes
3. Cecal intubation for all colonoscopies: 90%

Data were extracted from the tableau with autoimport feed function from the ProVation database. The data were transferred to SPSS (IBM Corp., released 2013, IBM SPSS Statistics for Windows, Version 22.0, Armonk, NY: IBM Corp.) and providers' information was deidentified.

The mean values of each metric from January through June (1^st^ half) and July through December (2^nd^ half) were compared using the paired Student *t*-test. We used curved estimation test to determine if the ADRs across the time periods (year-wise and month-wise) were statistically significant or random events in our series.

### 2.1. Statistical Analysis

Mean changes in outcomes across study time periods were examined using a series of paired *t*-tests for all possible pairwise comparisons for each corresponding year. Analyses were conducted in SPSS (IBM Corp., released 2013, IBM SPSS Statistics for Windows, Version 22.0, Armonk, NY: IBM Corp.), and all statistical tests assumed a 5% level of significance.

## 3. Results

The electronic endoscopy suite data contained information for 28–30 providers over the years 2011–2015. Based on the inclusion criteria, 15 providers who performed more than 20 colonoscopies per year in all 5 years were eligible for the study.

### 3.1. Adenoma Detection Rate (ADR)

**Figure 1:**
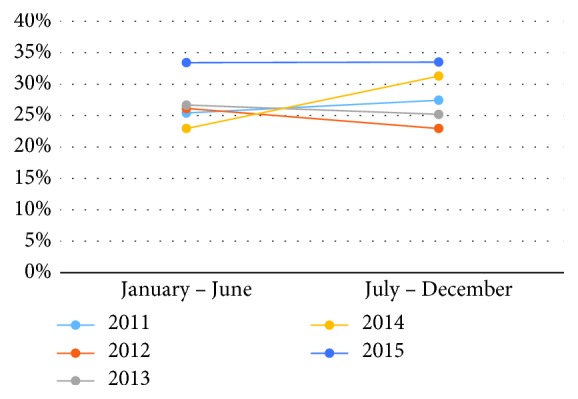
Comparison of adenoma detection rate (ADR) in January-June to July-December.

The collective ADR of all providers in the first half of all years was 26.9% while in the 2^nd^ half it was 28.1% (*p* = 0.476). The distribution of the ADR in the individual years is presented in Table 1. The ADR decreased in the 2^nd^ half of 2012 (26.1% to 22.9%) and 2013 (26.7% to 25.2%) as compared to the first half whereas it increased in the remaining years. However, there was no significant difference in the collective ADR comparing both time periods.  Figure 1 represents the data per year.

The collective ADR of all providers for female patients in the individual years was above 15%, which was the national benchmark for ADR for female patients until 2015 [7]. The collective providers' ADR for individual years is presented in Table 2. There was an increase in the ADR for female patients in the years 2011 (21.9% to 26.5%, *p* = 0.336) and 2014 (18.8% to 22.5%, *p* = 0.258) but was not significant. For the years 2012, 2013, and 2015, the ADRs decreased in the second half but were maintained over 15%.

**Figure 2:**
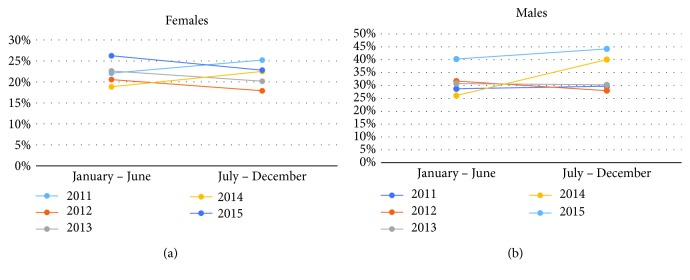
ADR for all providers for female (a) and male (b) patients.

For male patients, collective ADR of all providers in the individual years was above 25%, which was the national benchmark for the ADR until 2015 [7]. The collective providers' ADR for individual years is presented in Table 2. The ADR increased in the last 2 years of the study (2014 and 2015) in the second half following the distribution of the report cards while they decreased in the second half in the first 3 years of the study. However, the ADR remained above 25% and the difference was not significant.  Figure 2 represents the data according to gender for each year of the study.

**Figure 3:**
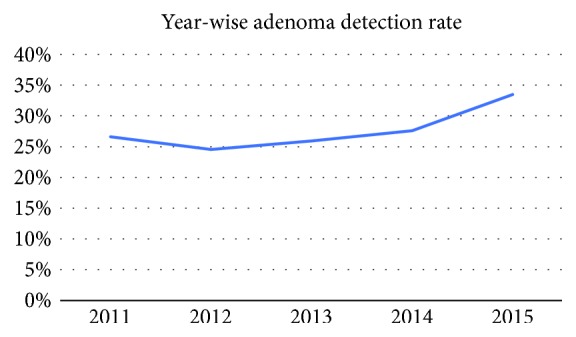
ADR trend over years for all providers.

The collective ADR for individual providers for all years combined is presented in Table 3 and is pictorially presented in Figure 3. Three providers (E, H, and M) were underperforming (<20%) in the first half, but the ADRs increased above 20% (the pre-2015 benchmark) in the second half following the report cards. For the remainder of the providers (*n* = 12) with ADRs above 20%, 8 providers had an increase in the ADR in the 2^nd^ half of the year following the report cards while 4 providers had a decrease in the ADR. However, these figures were maintained above national benchmark standards.

**Figure 4:**
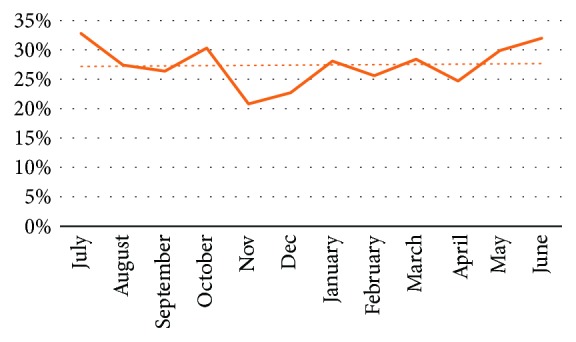
Trend of adenoma detection rate using curve estimation test from 2011 to 2015.

There was an increasing trend seen in the ADR among all the providers from the year 2011 to 2015; however, this trend was not statistically significant. The data are presented in Table 4 and Figure 1, respectively. Month-wise distribution of the ADR from July 2011 to December 2015 did not show any significant change. These data are presented in Figure 4.

### 3.2. Cecal Intubation

All providers exceeded national benchmark rates for CI from the year 2011 to 2015, i.e., >90%. We performed individual explorative analyses similar to the ADR and found no statistical change in the CI for any providers following the distribution of the report cards.

For individual providers, the CI data for all years (2011–2015) is presented in Table 5. There have been variations in the trends of the CI following the report cards, but none of these figures meet statistical significance.

### 3.3. Colonoscope Withdrawal Time

All providers, except one, had higher than national benchmark standards for CW from the year 2011 to 2015 (i.e., minimum 6 minutes) [10]. The collective CW of all providers in the first half of all years was 10.4 minutes while in the 2nd half it was 10.8 minutes (*p* = 0.285). The distribution of the CW in the individual years is presented in Table 6. The CW increased in the 2nd half of all years, except 2011, as compared to the first half. However, there was no significant difference in the collective CW.

**Figure 5:**
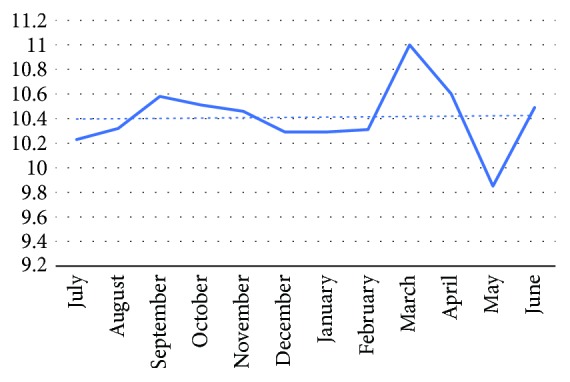
Trend for colonoscope withdrawal time month-wise from 2011 to 2015.

Similar to the ADR, month-wise distribution of the CW from July 2011 to December 2015 did not show any significant change. These data are presented in Figure 5.

## 4. Discussion

Based on the results of our study, we found that report cards distributed in the middle of the year do not improve outcomes among adequately performing providers, who were performing on par with the national benchmark standards. These report cards may, however, help maintain quality performance.

To decrease the mortality of colorectal cancer by colonoscopy requires the ability to detect adenomas. To decrease the miss rates owing to underperformance, guidelines have been established. National standard benchmarks were devised based on observational studies to increase the effectiveness of colonoscopy. Adenoma detection rate (ADR), colonoscope withdrawal time (CW), and cecal intubation (CI) were considered as the most important quality indicators. Based on these indicators, institutions may distribute report cards to endoscopists with these details.

Kahi et al. demonstrated an increase in ADR (44.7% to 53.9%) and CI (95% to 98.1%) at the VA Medical Center associated with Indiana University with quarterly distributed report cards [14]. In our study, the overall ADR increased from the year 2011 (26.6%) to 2015 (33.5%); however, the trend was not statistically significant. The ADR for individual years in the second half for all the providers oscillated around the ADR for the first half but was maintained above the national benchmarks. Three providers had ADR less than 20% in the first half of the years (collectively) increasing in the second half of the years (E, H, and M) following the distribution of the report cards. Since the report cards are distributed in an anonymous manner, we believe that the report cards were responsible for improving the ADR for these 3 underperforming endoscopists. We hypothesized that the months following the distribution of the report cards (e.g., July through December) would result in a transient increase in the ADR. There was no difference in the ADR following the month of June, and no trend was seen in the subsequent months. The report cards have now been revised, from the year 2016, to the more recent update by the joint task force of the American College of Gastroenterology and the American Society of Gastrointestinal Endoscopy which recommended ADR benchmarks of 25% for all patients and sex-specific rates of 30% for male patients and 20% for female patients after analyzing [17].

Prior studies have shown a significant increase in the CW from 6.57 minutes to 8.07 minutes [16], while some studies show no change [15]. The average CW in our study was 10.4 in the first half and 10.8 minutes the second half. There was a slight increase in the CW, but since the endoscopists were performing beyond the national benchmarks, there was no significant change.

Strengths of our study include the length of analysis, encompassing a continuous 5-year period. All 15 providers have been at this institution for the period of the study including at the time the report cards began being distributed. Since 2011, we have had a strong monitoring and feedback program as it pertains to maintaining quality colonoscopy. Our study had certain limitations. We could not exclude patients who had a prior colonoscopy at an outside institution, so we could not be sure if patients' screening colonoscopy was their first such examination. We did not have access to risk factor data, such as a personal or family history of CRC, a previous colon resection, inflammatory bowel disease, or polyposis syndrome, which would impact adenoma prevalence. Moreover, it is not clear whether the report card strategy would be effective in other settings, with a larger group of endoscopists or with a more diverse patient population. Finally, most of the endoscopists in our study were high performing at baseline; thus, whether report cards would benefit endoscopists with low-level adenoma detection rates remains speculative and requires further study.

## 5. Conclusion

Midyear reporting of colonoscopy quality indicators is not associated with a transient increase in the quality measures in already high-performing experienced endoscopists. However, it is plausible that report cards may help in maintaining the metrics in this population. Our study shows that report cards may possibly be beneficial to providers with lower than national benchmark metrics. This effect should be explored further with a larger series over a longer period of time.

## Figures and Tables

**Table 1:** Comparison of ADR in January-June to July-December.

Years	January–June	July–December	*p* value
2011	25.7%	27.5%	0.713
2012	26.1%	22.9%	0.384
2013	26.7%	25.2%	0.682
2014	25.4%	29.8%	0.162
2015	33.4%	33.5%	0.983
All (2011-2015)	26.9%	28.1%	0.476

**Table 2:** ADR for all providers for female and male patients.

	Years	January–June	July–December	*p* value
Females	2011	21.9%	26.5%	0.336
	2012	20.56%	17.9%	0.406
	2013	22.6%	20.2%	0.476
	2014	18.8%	22.5%	0.258
	2015	26.2%	22.8%	0.433

Males	2011	29.4%	28.6%	0.898
	2012	31.7%	28.0%	0.587
	2013	30.7%	30.2%	0.931
	2014	31.9%	37.1%	0.357
	2015	40.6%	44.2%	0.579

**Table 3:** ADR for 15 providers for the 1^st^ half (2011–2015) and the 2^nd^ half (2011–2015).

Providers	January-June	July-December	*p* value
A	32%	24%	0.346
B	29%	22%	0.347
C	36%	20%	0.129
D	25%	28%	0.378
E	19%	26%	0.114
F	35%	46%	0.111
G	31%	33%	0.794
H	19%	24%	0.357
I	28%	24%	0.704
J	24%	27%	0.252
K	29%	32%	0.352
L	28%	31%	0.507
M	15%	21%	0.085
N	31%	35%	0.558
O	21%	25%	0.611

**Table 4:** ADR trend over years for all providers.

Years	ADR	*p* value
2011	26.60%	0.406
2012	24.53%	
2013	25.94%	
2014	27.6%	
2015	33.47%	

**Table 5:** Cecal intubation for 15 providers for the 1^st^ half (2011–2015) and the 2^nd^ half (2011–2015).

Providers	January–June (%)	July–December (%)	*p* value
A	92.6	99.2	0.103
B	93.8	97.8	0.245
C	93.2	95.6	0.114
D	96.7	97.1	0.497
E	98.8	99.0	0.8
F	100.0	99.0	0.242
G	98.6	97.0	0.617
H	98.6	98.0	0.672
I	98.3	98.5	0.85
J	99.9	99.2	0.111
K	98.5	98.5	0.946
L	99.0	99.0	0.966
M	99.2	99.5	0.391
N	98.7	98.8	0.925
O	94.0	91.1	0.664

**Table 6:** Colonoscope withdrawal time (in minutes) for 15 providers for the 1^st^ half (2011–2015) and the 2^nd^ half (2011–2015).

CW in minutes	January-June	July-December	*p* value
2011	10.6	10.1	0.407
2012	10.0	10.5	0.111
2013	10.9	12.1	0.293
2014	10.4	10.7	0.233
2015	10.3	10.6	0.264
All years	10.4	10.8	0.285

## Data Availability

The retrospective data used to support the findings of this study may be released upon application to the Institutional Review Board of Columbia University.
